# Minimal Associations Between Clinical Data and Children's Self-Reported Health-Related Quality of Life in Children With Chronic Conditions—A Cross-Sectional Study

**DOI:** 10.3389/fped.2019.00017

**Published:** 2019-02-05

**Authors:** Kathrin I. Fischer, Dana Barthel, Christiane Otto, Ulrike Ravens-Sieberer, Ute Thyen, Marcus Klein, Otto Walter, Matthias Rose, Sandra Nolte

**Affiliations:** ^1^Charité – Universitätsmedizin Berlin, Corporate Member of Freie Universität Berlin, Humboldt-Universität zu Berlin, and Berlin Institute of Health, Medical Department, Division of Psychosomatic Medicine, Berlin, Germany; ^2^Research Unit Child Public Health, Department of Child and Adolescent Psychiatry, Psychotherapy, and Psychosomatics, Center for Psychosocial Medicine, University Medical Center Hamburg-Eppendorf, Hamburg, Germany; ^3^Department of Pediatric and Adolescent Medicine, Universität zu Lübeck, Lübeck, Germany; ^4^Department of General Pediatrics, Christian-Albrechts-Universität, Kiel, Germany; ^5^Department of Quantitative Health Sciences, University of Massachusetts Medical School, Worcester, MA, United States; ^6^Public Health Innovation, Population Health Strategic Research Centre, School of Health and Social Development, Deakin University, Geelong, VIC, Australia

**Keywords:** health-related quality of life, pediatrics, self-report, patient outcome assessments, chronic disease, computer-adaptive testing

## Abstract

**Introduction:** The improvement—or at least maintenance—of health-related quality of life (HRQoL) in children and adolescents is one of the main aims of chronic disease care. This study examines HRQoL of children and adolescents with three different chronic conditions (i.e., diabetes mellitus, asthma, juvenile arthritis) using the computer-adaptive test Kids-CAT, comprising five HRQoL domains: physical well-being, psychological well-being, parent relations, social support and peers, and school well-being. Further, associations between HRQoL and distinct clinical data and medical assessments are investigated to explore how much variability of the five domains can be explained by these variables.

**Methods:** Cross-sectional data of the Kids-CAT study was analyzed. The Kids-CAT was used in two outpatient clinics in northern Germany gathering data on self-reported HRQoL in *n* = 309 children and adolescents aged 7–17 years. Additionally, general patient information, clinical data, and pediatrician-reported medical assessments were measured. Multiple regression analyses were conducted to explore associations between HRQoL and selected variables (i.e., disease duration, co-morbidity, disease control, overall health status).

**Results:** Overall, self-reported HRQoL in all five domains were comparable to data of an age- and sex-matched reference population. Results of regression analyses indicated that the investigated variables only minimally explain variance in the five Kids-CAT domains. Sociodemographic, clinical data, and medical assessments explained 18.4% of the variance in physical well-being, 10.7% in psychological well-being, and < 10% of the variance in parent relations, social support and peers, and school well-being.

**Conclusion:** Sociodemographic data, disease duration, co-morbidity, and medical assessments, such as disease control or pediatrician-assessed overall health status show low association with HRQoL of children and adolescents with chronic conditions. Data on self-reported HRQoL delivers valuable information on children's well-being and can improve healthcare professionals' understanding of the subjective well-being of their young patients. The implementation of tools like the Kids-CAT can facilitate the identification of potential problem areas, which should enable healthcare professionals to better address specific healthcare needs.

**Clinical Trial Registration:** identifier: DRKS00006326 (retrospectively registered); Date of registry: August 1st, 2014.

## Introduction

An increasing number of children and adolescents live with chronic conditions, such as asthma, diabetes mellitus or juvenile arthritis (1, 2). The aim of healthcare is to improve clinical outcomes of these young patients. Over the past few decades, advancements in healthcare have resulted in new and advanced treatment modalities that led to a reduction of symptoms, improved survival, and increased life expectancy among children and adolescents with chronic disease (1, 3). As such improvements lead to an overall increase in years lived with disease, health-related quality of life (HRQoL) is an increasingly important outcome in healthcare (2, 4).

The concept of HRQoL, as defined by the World Health Organization, is “a broad ranging concept affected in a complex way by the person's physical health, psychological state, level of independence, social relationships, and their relationships to salient features of their environment” (5). In medical research, HRQoL has become an important outcome measure over the last decades, while it is also increasingly recognized in clinical practice as a valuable and important source of information, especially regarding patient-centered care (6–8).

The assessment of HRQoL in clinical practice has the potential to support healthcare in various ways. Patient-reported outcome (PRO) measures can be applied and used in various ways, e.g., as screening or monitoring tools, to promote patient-centered care or to facilitate communication on an individual patient level (9). The implementation of PRO measures can enable clinicians to identify problems in specific HRQoL domains and additional healthcare needs, which might not be detected otherwise (10, 11). Overall, the assessment of HRQoL supports healthcare professionals to get a more comprehensive idea of patient's health status (8, 10, 11).

While various generic and disease-specific HRQoL instruments are available for use in pediatrics (12), widespread implementation of HRQoL instruments in clinical practice has not yet been accomplished (13). Previous research identified various obstacles of implementing HRQoL measures in clinical practice, such as the additional effort in proportion needed (8, 14, 15). In recent years, technical and methodological advancements have led to a new generation of HRQoL instruments, i.e., computer-adaptive tests (CAT). CATs are developed based on modern test theory methods and their dynamic nature have the potential to increase measurement precision while reducing respondent burden (16). The electronic assessment also reduces staff burden compared to paper-pencil assessment of HRQoL. Through electronic assessment data is automatically stored in a data base and immediately scored, so that results are available in real-time (15, 16). These features of CATs might reduce logistical barriers. To our knowledge, only few CATs are available in the field of pediatrics. Besides the PEDI-CAT, a revised version of the Pediatric Evaluation of Disability Inventory (PEDI) (17), the CP-CAT, used to measure physical functioning in children with cerebral palsy (18), and the Kids-CAT, a generic instrument to assess HRQoL in children (19, 20), have been developed in pediatric care. Further, the PROMIS initiative developed a number of pediatric item banks available for CATs (21).

Despite recent advancements in the field of patient-reported outcomes with the development of increasingly sophisticated assessment instruments, many clinicians still assume that additional information regarding relevant problems of their patients are not needed, as these issues would be discovered during the consultation (8). Generally, outcome measures predominantly focus on clinical parameters, such as symptoms and laboratory diagnostics to monitor and evaluate healthcare. Combined with patients' medical history and medical assessments during the consultation, these parameters help treating pediatricians to gain a picture of the patients' health status and are the basis for treatment decisions. Most commonly, if assessed at all, HRQoL is assessed in a rather unstandardized way during or after the consultation, e.g., pediatricians' proxy estimation of overall health of the young patient (11).

This raises the question whether the available information is sufficient for pediatricians to get a precise picture of patients' health status or whether the compilation of information that is usually available to pediatricians bears the risk of misjudgment of their patients' HRQoL if HRQoL outcomes are not routinely assessed. We hypothesized that there is only little relation between HRQoL outcomes, and factors routinely assessed in pediatric care.

The aim of this study was (1) to estimate HRQoL of children and adolescents with chronic conditions in relation to a reference population and (2) to explore how much variability of HRQoL domains could be explained by sociodemographic factors, selected pediatrician-reported clinical data, and pediatricians' medical assessments of their young patients.

## Materials and Methods

The STROBE guideline was used for reporting study results (22). The STROBE statement checklist can be found in the Supplementary Material (Supplement Table 2).

### Study Design, Setting, and Participants

Cross-sectional data (baseline assessment) of the Kids-CAT study, a prospective longitudinal observational study, is used (20). Data was collected at two specialist outpatient clinics at the University Medical Center Schleswig Holstein in Kiel and Lübeck, Germany, between June 2013 and April 2014. Study nurses recruited children and adolescents, attending the clinics for regular check-ups (convenience sample). Eligibility criteria were age (7–17 years), clinical diagnosis of asthma, diabetes mellitus, or juvenile arthritis [according to International Classification of Disease, 10th version, German modification (23)], sufficient reading skills, and sufficient knowledge of German, (assessed through parents). Out of 397 patients approached by the study nurses, 312 children and adolescents participated in the study (24). Three cases were excluded from the presented analyses due to missing HRQoL data; hence, *n* = 309 children and adolescents were included in this study. The overall study design and process of recruitment is described elsewhere (20).

### Outcome Variables—Self-Reported Health-Related Quality of Life

Self-reported HRQoL was assessed using the Kids-CAT, one of the first CATs in the field of pediatrics (19). It is based on the domain structure of the KIDSCREEN-27 (25) and covers the five domains physical well-being, psychological well-being, parent relations, social support and peers, and school well-being. The Kids-CAT is constructed on the basis of item response theory (26, 27). Each domain consists of an underlying item bank, including various items to measures a wide range of the respective domain. The Kids-CAT comprises in total 155 items. The item bank physical well-being consists of 26 items, 46 items are included in the psychological well-being item bank, the item banks parent relations and social support and peers comprises 26 items, and 31 items are included in the item bank to assess school well-being of children and adolescents. Due to the adaptive nature of CATs, children have to answer a maximum of 35 items, as specified in the stopping rules (maximum seven items per domain to be answered, or measurement precision of 0.95 is reached). Computerized adaptive testing works based on a CAT algorithm administering individual item sets to each child/adolescent. Once all items are filled out by the child, the respondent's latent trait scores (theta scores) are computed for each domain providing an estimation of the value of the assumed latent construct, i.e., physical well-being, psychological well-being, parent relation, social support and peers, and school well-being. Data from four large norm studies, including ~10,500 children and adolescents of German-speaking countries (Austria, Germany, Switzerland) was merged and used to calibrate the five item banks applying the KIDSCREEN items as anchors (19). Theta scores were transformed to a T-scores metric (mean *M* = 50 and standard deviation, *SD* = 10), with a score of 50 representing the mean score of a sex-and age-matched reference population (28). In this way, Kids-CAT scores are set in relation to the reference population and can be interpreted accordingly. The mean age of the reference population was between 12.8 and 13.3 years depending on the domain and ~ 6% had a chronic disease or disability (19). The development process of the Kids-CAT is reported elsewhere (19). Results from validation study suggest satisfactory psychometric properties of the Kids-CAT (20). Further, the Kids-CAT appears to be feasible and acceptable in clinical practice as reported by physicians, children, and adolescents (24).

### Predictor Variables—Pediatrician-Reported Clinical Data and Medical Assessments

Pediatricians were asked to complete a form for all participating children and adolescents within or after patient consultation, including the following items: year of diagnosis, co-morbidity, disease control, and pediatricians' assessment of patients' overall health status.

We estimated disease duration based on the year of diagnosis. For additional analysis purposes, we transformed the continuous variable disease duration into a dichotomous variable based on a median split with the categories “less than or equal to 5 years” and “more than 5 years.” Co-morbidity was assessed using an open-ended question to be completed by the treating pediatrician considering additional diagnoses (according to International Classification of Disease, 10th version, German modification (ICD-10-GM) (23). We created a binary variable indicating whether at least one additional chronic health condition was diagnosed.

Disease control was assessed using a set of disease-specific items for the three chronic conditions. For asthma, items regarding symptoms during day and night, restrictions in daily life activities, use of emergency medication, information on lung function, and occurrence of exacerbation were assessed (29). For diabetes, information on HbA1c-level, existence of vascular complications, existence of further autoimmune diseases, occurrence of ketoacidosis over the past 4 weeks, and hypo- or hyperglycemic episodes requiring further treatment were assessed (30). For juvenile arthritis, items were based on the International League of Associations for Rheumatology (ILAR) classification and included assessment of current disease activity level, questions about mobility, and interference with eyes (31). Based on medical guidelines and consensus between clinicians and researchers, disease-specific sum scores were computed and then classified into good vs. poor disease control. For asthma the score ranged from zero to seven (cut off score: >0), for diabetes from 0 to 12 points (cut off score: >1) and for juvenile arthritis from three to eight (cut off score: >4). For all disease –specific sum scores a higher score indicated worse disease control. For more details, see Supplement Table 1.

Further, we asked pediatricians to rate the overall health status of their patients by use of a 5-point Likert scale ranging from very good to very poor. For further analyses, we created a dichotomous variable by collapsing the categories very good and good and the categories fair, poor, and very poor. However, the latter category was not chosen by any of the treating pediatricians.

### Predictor Variables—Sociodemographic Characteristics

Age and sex of participants were extracted from medical records. For sub-analysis, age was grouped into the two categories children (7–11 years) and adolescents (12–17 years).

Following the approach by Lampert et al. information on socioeconomic status (SES) for both parents were assessed using eight validated questions on education, occupation, and income (32). Weighted sum scores (3–21 points) of these items were computed and further categorized into low (3–7.9 points), medium (8–13.8 points), and high SES (13.9–21 points) (32, 33).

### Data Analyses

Descriptive analyses for sociodemographic characteristics, pediatrician-reported clinical data and medical assessments were conducted including bivariate analyses to compare the three disease-specific groups of our sample. Analyses of variance (ANOVA) were performed with robust option Brown-Forsythe (34) for continuous variables (i.e., age, disease duration) and χ^2^-tests for categorical variables (i.e., sex, age group, SES, co-morbidity, disease control, overall health status).

To explore self-reported HRQoL, mean T-scores for the five Kids-CAT domains were calculated for the total sample as well as stratified by sociodemographic characteristics, disease group, pediatrician-reported clinical data and medical assessments. Analyses included independent *t*-tests, ANOVA, and χ^2^-tests.

Finally, five linear regression models were calculated. For these analyses, we aimed to use complete datasets. Out of 309 cases 73 cases (23.6%) presented incomplete data in six variables out of 13 variables included in our analyses. The variable SES (20.1%) presented a considerably higher percentage of missing values in our sample compared with other variables. For disease control, 3.6% of the values were missing. For all other variables the percentage of missing was <2%. As data is assumingly missing completely at random (MCAR), following Little's test (35) that resulted in a χ^2^ = 37.756 (df = 37; *p* < 0.435), we applied multiple imputations (MI) for missing data replacement using the fully conditional specification method to create *m* = 20 datasets (35, 36). For the linear regression models, socio-demographic variables, pediatrician-reported clinical data, and medical assessments were entered as independent variables, while the Kids-CAT domains were defined as outcomes, i.e., dependent variables. Each model was used to determine how much variation in the corresponding HRQoL domain could be explained by independent variables and if individual independent variables significantly predicted the dependent variable. The models included the following independent variables: age (in years), sex, disease group, and SES (sum score), disease duration, co-morbidity, disease control and overall health status rated by pediatricians. For disease group, dummy-coded variables were created with “juvenile arthritis” as reference group. To check the validity of the results, sensitivity analysis were conducted comparing the linear regression models using the pooled results of the imputed data sets to the results of the original data using complete case analyses.

Statistical analyses were performed using R Studio Version 1.0.136 with package mice (37) and IBM SPSS Statistics for Windows Version 22.0.

### Ethics Approval and Consent to Participate

All procedures performed were in accordance with the ethical standards of the institutional and/or national research committee and with the 1964 Helsinki declaration and its later amendments or comparable ethical standards. Ethical approval was obtained from the Chambers of Physicians Kiel and Lübeck, and Chamber of Psychotherapists Hamburg, Germany. Informed consent was obtained from all participants included in the study (informed consent of parents or legal guardians, informed assent of children and adolescents).

## Results

### Sample Characteristics

Cross-sectional data was available from 309 children and adolescents (Table 1). Of these, 18.8% (*n* = 58) were diagnosed with asthma, 65.4% (*n* = 202) with diabetes mellitus (type 1), and 15.9% (*n* = 49) with juvenile arthritis. Mean disease duration was *M* = 5.44 (*SD* = 3.73) years. At least one additional diagnosed health condition was reported for 32.5% (*n* = 100) of the participants. Based on pediatricians' assessments, disease control was rated as poor for 29.2% (*n* = 87) of the participants; overall health status was assessed by pediatricians as fair or poor for 19.1% (*n* = 58) of the children and adolescents.

**Table 1:** Sociodemographic variables, pediatrician-reported clinical data, and medical assessments of chronically ill children and adolescents.

	***N***	**Total sample**	**Asthma**	**Diabetes**	**Juvenile arthritis**
		**(*n* = 309)**	**(*n* = 58)**	**(*n* = 202)**	**(*n* = 49)**
		**Mean (SD) or %**	**Mean (SD) or %**	**Mean (SD) or %**	**Mean (SD) or %**
**SOCIODEMOGRAPHIC CHARACTERISTIC**					
Age in years, *mean (SD)*	309	12.49 (2.79)	11.76 (2.45)	12.74 (2.79)	12.29 (3.01)
Age group, %	309				
Children (7–11 years)	118	38.2	46.6	36.1	36.7
Adolescents (12–17 years)	191	61.8	53.4	63.9	63.3
Sex (female), %	148	47.9	37.9	45.0	71.4
Socioeconomic status of the family, %	247				
High	66	26.7	27.3	26.8	25.6
Medium	174	70.4	70.5	70.1	71.8
Low	7	2.8	2.3	3.0	2.6
**PEDIATRICIAN-REPORTED CLINICAL DATA AND MEDICAL ASSESSMENTS**					
Disease duration in years, *mean (SD)*	307	5.43 (3.72)	6.48 (3.10)	5.43 (3.74)	4.24 (3.99)
Co-morbidity (yes), %	308	32.5	86.0	19.8	22.4
Disease control, %	298				
Good	211	70.8	53.6	74.1	77.8
Overall health status (pediatrician assessment), %	304				
Very good / Good	246	80.9	59.6	87.4	79.2

### Self-Reported Health-Related Quality of Life

Mean T-scores for all Kids-CAT domains (Table 2) as well as associated 95% confidence intervals for the clinical sample were within the normal range of 40–60 as determined based on data of the reference population (with mean = 50).

**Table 2:** Self-reported HRQoL in groups according to sociodemographic and pediatrician-reported clinical data and medical assessments among chronically ill children and adolescents.

				**Physical well-being**	**Psychological well-being**	**Parent relations**	**Social support and peers**	**School well-being**
			***N***	***M (SD)***	***M (SD)***	***M (SD)***	***M (SD)***	***M (SD)***
Socio-demographic characteristics	Sex	Male	161	48.78 (9.72)	52.15 (8.57)^*^	53.73 (9.13)	53.77 (8.27)	52.44 (9.41)
		Female	148	47.48 (11.07)	47.57 (9.51)^*^	53.97 (8.74)	54.46 (8.19)	52.04 (9.70)
	Age group	Children	118	49.10 (9.47)	49.79 (9.08)	52.61 (8.75)	52.58 (8.57)^*^	51.94 (10.06)
		Adolescents	191	47.57 (10.90)	50.06 (9.46)	54.61 (8.98)	55.04 (7.88)^*^	52.45 (9.22)
	Disease group	Asthma	58	45.19 (11.66)^*^	48.91 (9.73)	52.72 (8.48)	54.95 (8.12)	52.13 (8.55)
		Diabetes	202	49.54 (9.54)^*^	50.23 (9.20)	53.91 (9.13)	54.33 (8.36)	52.03 (9.93)
		Juvenile Arthritis	49	45.96 (11.25)	50.08 (9.30)	54.90 (8.61)	52.16 (7.64)	53.32 (9.10)
	Socioeconomic status	High	66	48.56 (10.52)	50.93 (7.22)	54.98 (7.86)	54.47 (7.02)	55.36 (8.68)^*^
		Medium	147	48.81 (10.29)	49.85 (10.28)	53.71 (9.17)	53.79 (8.84)	50.88 (9.67)^*^
		Low	7	40.37 (14.11)	50.28 (9.69)	55.22 (6.38)	51.20 (7.62)	50.08 (9.74)
Pediatrician-reported clinical data and medical assessments	Disease duration	≤ 5 years	159	48.46 (9.83)	50.56 (9.25)	54.76 (8.71)	53.89 (8.79)	52.47 (9.75)
		>5 years	148	47.99 (10.83)	49.29 (9.40)	52.88 (9.08)	54.32 (7.61)	52.01 (9.39)
	Co-morbidity	Yes	100	46.09 (10.94)^*^	49.35 (9.73)	53.52 (8.82)	55.35 (7.91)	52.08 (9.08)
		No	208	49.27 (9.83)^*^	50.26 (9.12)	53.96 (9.00)	53.46 (8.32)	52.32 (9.79)
	Disease Control	Good	211	50.13 (9.52)^*^	50.89 (9.12)^*^	54.56 (9.08)	53.66 (8.26)	52.83 (9.76)
		Poor	87	44.40 (10.79)^*^	47.88 (9.13)^*^	52.50 (8.41)	55.26 (8.11)	50.93 (8.95)
	Overall health status	Very good / Good	246	50.02 (9.39)^*^	50.81 (8.80)^*^	54.55 (8.70)^*^	54.02 (8.04)	52.80 (9.50)^*^
		Fair / Poor	58	40.73 (10.85)^*^	46.38 (10.38)^*^	50.90 (9.29)^*^	54.10 (9.13)	49.86 (9.57)^*^
Total				48.15 (10.39)	49.96 (9.30)	53.84 (8.93)	54.10 (8.22)	52.25 (9.54)

* *p < 0.05, based on t-tests or analyses of variance (ANOVAs) with Hochberg's GT2 Post hoc test*.

**Figure 1:**
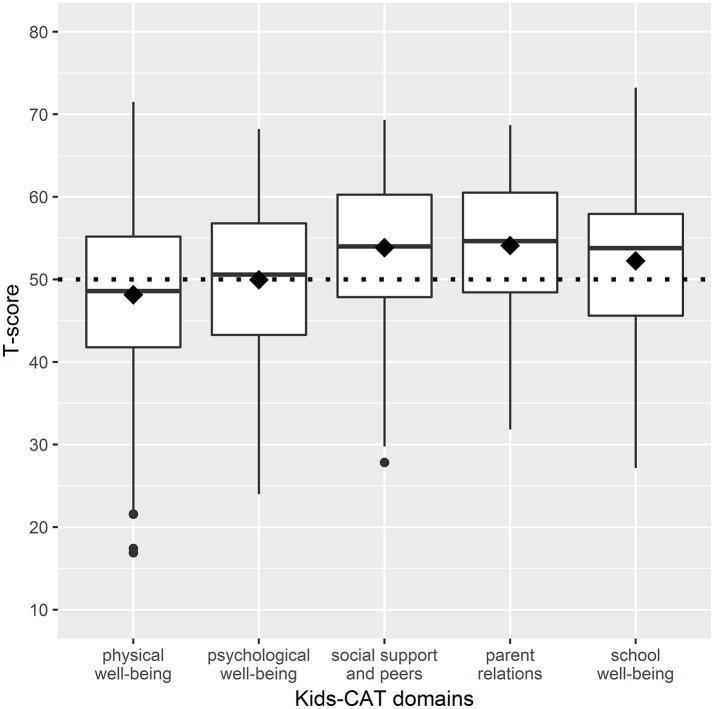
Boxplots of HRQoL dimensions - distribution of T-scores of the five Kids-CAT domains. The boxes represent the interquartile range between the first and the third quartile, the horizontal bold line in each box shows the median T-score of each Kids-CAT domain. The diamond shows the mean score of the respective domain. The small black dots beyond the end of the whiskers are outliers. The dotted line represents the mean T-score of the reference population (T-score of 50 with a SD of +/− 10).

Mean T-scores of the physical well-being domain were slightly lower for study participants compared to children/adolescents of the reference population. In contrast, T-scores reported for parent relations, social support and peers, and school well-being were slightly higher than mean scores of the reference population. However, our sample showed a wide distribution within domain scores with a range of 54.6 for physical well-being (*M* = 48.15; *SD* = 10.39), 44.2 for emotional well-being (*M* = 49.96; *SD* = 9.30), 41.49 for parent relations (*M* = 53.84; *SD* = 8.93), 36.86 for social support and peers (*M* = 54.10; *SD* = 8.22), and 46.09 for school well-being (*M* = 52.25; *SD* = 9.54) (Figure 1). Further analyses showed that 35.3% (*n* = 109) of children and adolescents indicated considerably lower HRQoL levels compared to the reference population in at least one of the five Kids-CAT domains, with respective T-score of < 40. Children and adolescents most frequently reported considerably lower physical well-being (21.4% (*n* = 66) of children and adolescents reported a T-score < 40).

Bivariate analyses (Table 2) revealed that differences in sociodemographic characteristics (i.e., sex, age group, SES, and disease group) were associated with significantly different scores in single Kids-CAT domains. Considering pediatrician-reported clinical data and medical assessments, statistically significant differences in physical well-being scores according to co-morbidity (*p* < 0.05), disease control (*p* ≤ 0.001), and overall health status (*p* ≤ 0.001) were found. Patients, whose overall health status had been rated as fair to poor by pediatricians, reported statistically lower physical well-being, psychological well-being, parent relations and school well-being compared to those who had been rated with very good to good overall health status.

### Associations Between Kids-CAT Domains and Clinical Data and Medical Assessment

The pooled results of 20 datasets following multiple imputation revealed that the proportions of variance in the five Kids-CAT domains that could be explained by sociodemographic variables, clinical data, and medical assessments was low (Table 3). For physical well-being, predictors explained 18.5% of the variance in the analyzed sample (*R*^2^ = 0.185, *adjusted R*^2^ = 0.160), whereas age, disease control, and fair to poor overall health status were statistically significant. For psychological well-being, 10.6% of the variance could be explained by the predictor variables (*R*^2^ = 0.106, *adjusted R*^2^ = 0.079), with sex and fair to poor overall health status being statistically significant. For parent relations, 4.7% of the variance could be explained (*R*^2^ = 0.047, *adjusted R*^2^ = 0.019) by the model, with age and fair to poor overall health status being statistically significant predictors of the model. For social support and peers, 6.5% of the variance could be explained (*R*^2^ = 0.065, *adjusted R*^2^ = 0.036), with age and SES being a statistically significant predictor. Finally, for school well-being, 6.3% of the variance could be explained (*R*^2^ = 0.063, *adjusted R*^2^ = 0.034), with SES as statistically significant predictor. Regarding the medical assessments of pediatricians, poor disease control correlated negatively with physical well-being. For overall health status, T-scores in the domains physical well-being, psychological well-being and parent relations were lower for patients with a fair to poor overall health status.

**Table 3:** Multivariable linear regression models of the relationship of sociodemographic variables and clinical data with the five Kids-CAT domains.

**Variable**	**Physical well-being**		**Psychological well-being**		**Parent relations**		**Social support and peers**		**School well-being**	
	**B (SE)**	**95%CI**	**B (SE)**	**95%CI**	**B (SE)**	**95%CI**	**B (SE)**	**95%CI**	**B (SE)**	**95%CI**
Constant	53.37		56.84		50.79		41.61		46.58	
Sex (female)	−0.60 (1.12)	[−2.80, 1.60]	−4.74^*^ (1.05)	[−6.81, −2.67]	−0.14 (1.03)	[−2.19, 1.91]	0.71 (0.95)	[−1.17, 2.58]	−0.64 (1.11)	[−2.82,1.54]
Age	−0.52^*^ (0.21)	[−0.93, 0.10]	−0.07 (0.20)	[−0.46, 0.32]	0.41^*^ (0.20)	[0.02, 0.80]	0.36^*^ (0.18)	[0.01, 0.72]	−0.07 (0.211)	[−0.48, 0.35]
Asthma^a^	1.28 (2.16)	[−2.97, 5.53]	−1.67 (2.03)	[−5.66, 2.31]	−0.92 (2.01)	[−4.87, 3.03]	1.78 (1.84)	[−1.83,5.40]	−0.97 (2.14)	[−5.18, 3.23]
Diabetes^a^	2.79 (1.57)	[−0.30, 5.87]	−1.31 (1.47)	[−4.19, 1.58]	−1.19 (1.46)	[−4.05, 1.68]	2.24 (1.33)	[−0.58, 4.86]	−1.42 (1.55)	[−4.46, 1.62]
SES^b^	0.08 (0.20)	[−0.32, 0.47]	−0.09 (0.19)	[−0.45, 0.28]	0.070 (0.21)	[−0.34, 0.47]	0.37^*^ (0.17)	[0.02, 0.71]	0.64^*^ (0.20)	[0.24, 1.04]
Disease duration	0.24 (0.16)	[−0.07, 0.56]	−0.07 (0.15)	[−0.36, 0.23]	−0.23 (0.15)	[−0.52, 0.07]	−0.02 (0.14)	[−0.29, 0.24]	−0.04 (0.16)	[−0.34, 0.27]
Co–morbidity	−1.40 (1.40)	[−4.17, 1.36]	0.10 (1.32)	[−2.48, 2.68]	0.29 (1.30)	[−2.27, 2.86]	1.85 (1.19)	[−0.49, 4.20]	0.19 (1.39)	[−2.55, 2.92]
Disease control (Poor)	−3.28^*^ (1.36)	[−5.99, −0.61]	1.28 (1.28)	[−3.80, 1.24]	−0.70 (1.25)	[−3.16, 1.76]	2.00 (1.16)	[−0.28, 4.29]	−0.47 (1.33)	[−3.08, 2.14]
Overall health status (Fair / Poor)	−7.15^*^ (1.57)	[−10.24, −4.06]	−3.68^*^ (1.50)	[−6.63, 0.73]	−3.15^*^ (1.46)	[−6.03, −0.28]	−0.99 (1.14)	[−3.63, 1.65]	−2.31 (1.55)	[−5.37, 0.75]
*R^2^ (adj. R^2^)*	0.19 (0.16)		0.11 (0.08)		0.05 (0.02)		0.07 (0.04)		0.06 (0.03)	
*ΔR^2^ (Δ adj. R^2^)*	0.12 (0.11)		0.03 (0.02)		0.03 (0.02)		0.02 (0.01)		0.01 (0.00)	

*n = 309 pooled data according to 20 imputed data sets*.

a *variables were entered as dummy variables with disease group juvenile arthritis used as a reference group*.

b Socioeconomic status (SES) was entered as continuous variable (sum score); B: unstandardized regression beta coefficient

* * p < 0.05; ΔR^2^: difference in R^2^ between basic model (sex, age, disease group, SES) and full model (sex, age, disease group, SES, disease duration, comorbidity, disease control, overall health status)*.

## Discussion

We found that children and adolescents with chronic conditions reported HRQoL scores that were on average comparable to an age- and sex-matched German-speaking reference population. That is, children and adolescents included in our sample assessed their HRQoL similarly to peers, even though they have a chronic condition and are undergoing routine medical treatment. While 21.4% of our sample indicated a considerably low physical well-being (i.e., T-score < 40), high scores were reported for parent relations and social support and peers. However, it should be noted that we did not compare HRQoL of chronically ill children to HRQoL of healthy peers, but made use of T-scores, which are centered to the scores of a relevant reference population. Previous studies that compared self-reports of chronically ill children to their peers without chronic conditions reported similar results for physical well-being, but they found lower scores for social domains, such as parent relations, social support and peers, and school well-being (25, 38). Similar to our findings, the difference in HRQoL scores between children with and without chronic conditions were also small for all measured health domains (25, 38). Considering that our data collection took place in an outpatient setting where children and adolescents attended the clinic for a regular check-up rather than attending because of acute symptoms, it is not surprising. Coping strategies and adaptation to illness could provide an explanation for our findings (1, 3). It should be further kept in mind that self-reported HRQoL measures how the young patients perceive their health status considering the individual limitations and opportunities (39, 40).

The comparison of T-scores according to sociodemographic and pediatrician-reported clinical data and medical assessment revealed statistically significant differences in specific domains. However, is should be noted that statically significant differences do not necessarily indicate clinically important differences (41). An interesting finding was the small positive effect of having at least one comorbid disease on the domains psychological well-being, parent relations, social support and peers, and school well-being. One reason for this finding could be additional support, care and attention from family and friends given the higher burden of having a comorbid disease.

When comparing HRQoL of children and adolescents with different health conditions, the characteristics of the underlying medical condition seems to influence the impact of the condition as also shown by others (3). Our data suggests that children and adolescents with diabetes mellitus reported the highest HRQoL scores followed by those with juvenile arthritis. Lowest scores, especially in the domain physical well-being, were reported by children and adolescents with asthma. Similar results were reported by Varni et al. (38) comparing HRQoL of children with different chronic conditions; however, in the Varni et al. study children and adolescents with juvenile arthritis reported worse HRQoL scores compared to those with asthma or diabetes mellitus (38). A viable explanation for the differences between our study and the Varni et al. (38) study may be explained by the high percentage of children and adolescents with asthma who have co-morbidities and poor disease control in our sample. Further, it might be related to the fact that, due to German national guidelines and the local conditions, children and adolescents with asthma are treated by their primary care pediatricians who only refer patients to specialized tertiary care clinics, if they do not achieve adequate disease control (42). Hence, it can be assumed that our asthma sample consisted only of those patients with the worst disease parameters. In contrast, patients with diabetes mellitus and juvenile arthritis are generally seen in specialized clinics; hence, our sample would have included the milder spectrum of patients of these two disease groups. This notion is reflected by the distribution of factor disease control. While half of the children and adolescents with asthma were allocated to the group “poor disease control”, less than one third were classified as poorly controlled for the diabetes mellitus and juvenile arthritis subsamples, respectively.

We found that selected predictor variables explained only little variability of the Kids-CAT domains. In particular, the proportions of variance explained by the investigated clinical data and medical assessments in the Kids-CAT domains were low ranging from 1.1% to 11.6%. Our findings can be explained by the model of patient outcomes by Wilson and Cleary (43). It conceptualizes the relationship of different outcomes in consideration of the somatic paradigm of health, reflecting that symptoms, functional status, and health perception are influenced by individual and environmental characteristics (43). While clinical data focuses on the underlying conditions and complaints of patients, the Kids-CAT domains refer to the functional status and health perception of young patients (43). The minimal association found in our study shows that both measures are linked but are clearly distinguishable from each other in terms of what they measure (44). Thus, it is hardly possible to draw inferences from clinical data and medical assessments to generic HRQoL of children and adolescents with chronic conditions and vice versa (45, 46). In particular, the only marginally explained variance for domain psychological well-being is an important result given the increased risk of children and adolescents with chronic conditions to develop emotional, developmental, and behavioral problems (11, 47). The implementation of a self-report instrument such as the Kids-CAT, with its feedback report on a domain level, enables pediatricians to gain crucial information about their young patients' HRQoL (24, 48, 49). It can be a useful adjunct to traditional clinical data to facilitate shared decision-making and support a patient-centered care approach (50).

Despite a comprehensive study design and thorough data collection, this study has potential limitations. First, data collection took place at two selected outpatient clinics and comprised participants recruited during regular check-ups. It turned out that the sample did not sufficiently cover children and adolescents with low socioeconomic status and immigrant background. Thus, findings based on our convenience sample are not generalizable. Second, both the selection and conceptualization of some of the variables may not have been optimal. Data on co-morbidity was reported by pediatrician and not directly obtained from the medical records. This might have resulted in a slight underreporting of additional medical conditions in the present study. Further, harmonizing the concept of disease control across three disease groups is a challenge and has been accomplished by a qualitative approach, which might be interpreted differently by other research groups. The selection, classification, and assessment of all clinical data was discussed thoroughly within the research group, including stakeholders with different research and clinical expertise. Finally, it should be noted that using the dichotomized variable for disease control represents a simplification of a complex construct, which has various characteristics in clinical practice.

## Further Research

Further research is needed to validate our results. On the one hand, further research is needed to test whether the additional information is beneficial for medical treatment. On the other hand, further research is needed to explore intra-individual variations of self-reported HRQoL as a measure of longitudinal adaptation and how this information can be incorporated into the treatment regimen over time. Moreover, it is important to define when a score is of clinical relevance and further action should be taken. Studies to determine minimal important difference using the Kids-CAT are needed for clinical guidance.

Given the advancements and increasing use of mobile applications to collect PRO data, it is possible to complete assessments almost everywhere. The impact of the setting should be addressed in further studies. In particular, it should be investigated how the setting (e.g., home assessments vs. assessment at the clinic) might influence the response behavior and thus the results for self-reported HRQoL in of children and adolescents.

## Conclusion

The variability of the HRQoL domains is only minimally explained by selected clinical data and medical assessments, indicating that children's HRQoL can hardly be inferred from these data alone. Especially, the minimal association between psychological well-being, and social health domains, such as parent relations, social support and peers, and school well-being and the selected clinical data shows that the assessment of HRQoL could be a valuable source of information in healthcare. Combining self-reported HRQoL with clinical parameters and traditional medical tests should enhance getting a more comprehensive picture of young patients' health status. In this way, pediatricians might identify additional healthcare needs, which would not be detected otherwise.

## Data Availability Statement

The raw data supporting the conclusions of this manuscript will be made available by the authors, without undue reservation, to any qualified researcher on request.

## Author Contributions

UR-S, MR, CO, UT, and MK contributed to study concept/design. OW, SN, CO, MR, and UR-S developed the Kids-CAT tool. DB, SN, UT, and MK supervised and managed data collection. OW, CO, and DB were responsible for data surveillance/quality and managed the data preparation. SN was involved in the data interpretation and conceptualization of the paper. KF performed statistical analyses and wrote the first draft of the manuscript. All authors critically revised the manuscript and approved the final manuscript as submitted.

### Conflict of Interest Statement

The authors declare that the research was conducted in the absence of any commercial or financial relationships that could be construed as a potential conflict of interest.

## Abbreviations

CAT: computer-adaptive test
HRQoL: health-related quality of life
SES: socioeconomic status.
