# Supplementary material for: Minimal Associations Between Clinical Data and Children's Self-Reported Health-Related Quality of Life in Children With Chronic Conditions—A Cross-Sectional Study
Source: Front Pediatr. 2019 Feb 5;7:17. doi: 10.3389/fped.2019.00017 (PMC6370724; doi:10.3389/fped.2019.00017)
Supplement: Supplementary file 1 [file Table_1.docx]

**Supplement Table 1**: Overview of variable disease control by disease group

**Asthma**

| *Item* | | *Response category* |
| --- | --- | --- |
| 1 | Asthma Symptoms during the daytime | no =0  yes =1 |
| 2 | Limitations in activities of daily living due to Asthma | no =0  yes =1 |
| 3 | Asthma Symptoms during nighttime / or awaking because of Asthma | no =0  yes =1 |
| 4 | Intake of PRN medication or emergency measures | no =0  yes =1 |
| 5 | Lung function | normal =0  <80% of FEV or PEF =1 |
| 6 | Occurrence of exacerbation during the last year | none =0  one or more per year =1  one per week =2 |
|  | Disease control (based on sum score) | good =0  poor >0 |

**Diabetes mellitus (type 1)**

| *Item* | | *Response category* |
| --- | --- | --- |
| 1 | HbA1c-value (%) (latest result) | <7.5% =0  7.5 -9.0% =1  >9.0% =2 |
| 2 | Existing vascular complications | none =0  yes, one =1  yes, at least 2 =2 |
| 3 | Additional autoimmune diseases requiring specific dietary or medication | none =0  yes, one =1  yes, at least 2 =2 |
| 4 | Diabetic ketoacidosis during the past 4 weeks | none =0  yes, one =1  yes, at least 2 =2 |
| 5 | Hyperglycemia requiring emergency measures during the past 4 weeks | none =0  yes, one =1  yes, at least 2 =2 |
| 6 | Hypoglycemia requiring emergency measures (glucagon or glucose (intravenous)) during the past 4 weeks | none =0  yes, one =1  yes, at least 2 =2 |
|  | Disease control (based on sum score) | good ≤1  poor >1 |

**Juvenile Arthritis**

| *Item* | | *Response category* |
| --- | --- | --- |
| 1 | ILAR classification | Polyarthritis (RF(+/-)) =2  all other forms or arthritis =1 |
| 2 | Ophthalmologic complications | no =1  yes =2 |
| 3 | Mobility impairments | none =1  occasionally limited =2  slightly limited =3  moderately to strongly limited =4 |
|  | Disease control (based on sum score) | good ≤4  poor >5 |
